# Exocytotic pore in a SNARE

**DOI:** 10.18632/oncotarget.17511

**Published:** 2017-04-28

**Authors:** Jernej Jorgačevski, Marko Kreft, Robert Zorec

**Affiliations:** Laboratory of Neuroendocrinology, Molecular Cell Physiology, Faculty of Medicine, University of Ljubljana, Ljubljana, Slovenia and Celica Biomedical, Ljubljana, Slovenia

**Keywords:** regulated exocytosis, fusion pore, fusion pore stability

**Figure 1:**
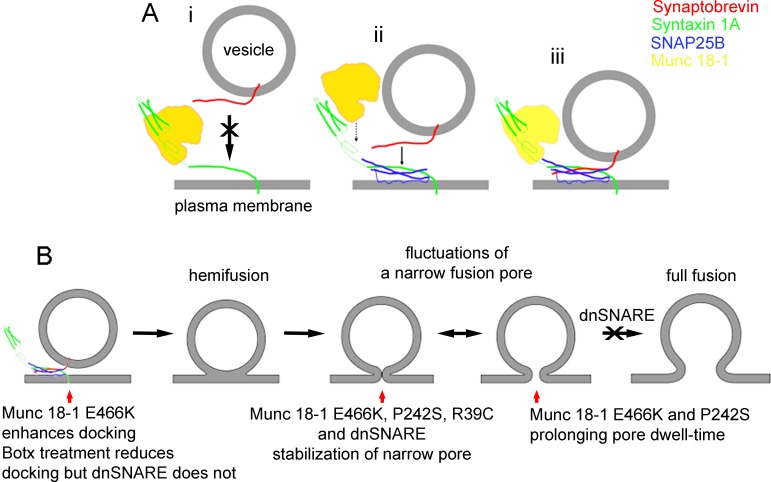
**A.** Munc18-1 favours the formation of the ternary SNARE complex, whereas synaptobrevin binds preferably with the binary complex between syntaxin and SNAP25. **B.** Transition of narrow fusion pores into full fusion is inhibited by dnSNARE peptides.

In 2013, the Nobel prize was awarded for discoveries related to the regulation of the cellular transport system (https://www.nobelprize.org/nobel_prizes/medicine/laureates/2013/). In addition to studies by Südhof's lab of signals that tell secretory vesicles when to release their cargo and the work of Schekman's lab describing a set of genes required for vesicle transport, the Rothman's lab determined the proteins termed SNAREs (soluble NSF-attachment protein receptors), that allow vesicles to fuse with their targets and thus transfer materials. Although the three interacting SNARE proteins (vesicle-associated membrane protein [VAMP]/synaptobrevin, synaptosome-associated protein of 25 kDa [SNAP-25] and syntaxin) had previously been identified by several scientists, and were localized to the presynaptic region, their function was largely unknown. VAMP/synaptobrevin was found to reside on the vesicle, whereas SNAP-25 and syntaxin were found at the plasmalemma. This led to the SNARE hypothesis, which stipulated that target and vesicle SNAREs were critical for vesicle fusion through a set of sequential steps of synaptic docking, activation and fusion of vesicle with the plasmalemma. This interpretation was influenced by bulk biochemical studies, which revealed that the ternary SNARE complex is a thermally stable structure [1], meaning that once the ternary SNARE complex is formed, its disassembly may take a long time, unless special enzymes are in action [2]. Moreover, these studies also implied that SNARE complex formation is associated with the vesicle membrane merger with the plasmalemma, enabling the vesicle cargo to be released in an all-or-none fashion, as originally considered by B. Katz. However, studies using atomic force spectroscopy of single molecule interactions revealed the disassembly properties of the ternary SNARE complex are occurring in the time domain of 0.2-2 s [3]. Therefore, assembly/disassembly of the ternary SNARE complex, influenced by SNARE accessory proteins, may take place not only at vesicle docking, facilitating the vesicle membrane merger with the plasmalemma, but also at other exocytotic stage intermediates (Figure 1), such as transient fusion pore widening and fusion pore dwell-time regulation, leading to full-fusion, a complete integration of vesicle membrane into the plasmalemma [4].

When the function of the accessory SNARE protein Munc18-1 was studied at the level of a single vesicle by the high-resolution capacitance technique, an ideal approach to study the membrane merger between a single vesicle and the plasmalemma [5], it was revealed that there may be multiple sites where the SNARE complex may play a role along the exocytotic intermediates [6]. In this study, Munc18-1 mutants were transfected into secretory cells to affect the interaction of Munc18-1 with syntaxin1 (Synt1) (R39C), Rab3A (E466K), and Mint proteins (P242S). In comparison with wild-type Munc18-1, mutant Munc18-1E466K increased the frequency of the unitary fusion events, consistent with the view that Rab3A protein facilitates vesicle docking at the plasmalemma. While the other Munc18-1 mutants (R39C and P242S) increased the fusion pore dwell-time, all the mutants stabilized a narrow fusion pore geometry, preventing transitions into a more widely or fully open one. Single-molecule atomic force microscopy experiments revealed that wild-type Munc18-1, but not Munc18-1R39C, abrogates the interaction between synaptobrevin2 (Syb2) and Synt1 binary *trans* complexes [6]. Importantly, neither form of Munc18-1 affected the interaction of Syb2 with the preformed binary *cis*-Synt1-SNAP25 complexes, revealing that Munc18-1 performs a proofing function by inhibiting tethering of Syb2-containing vesicles solely to Synt1 at the plasmalemma and promoting vesicular tethering via Syb2 to the preformed binary *cis* complex of Synt1-SNAP25 (Figure 1A).

The last transition prior to full-fusion is inhibited by the dominant-negative domain of synaptobrevin 2 protein peptide (dnSNARE, Figure 1B). This peptide has been considered to block the formation of the ternary SNARE complex and was hence used to block vesicle-based exocytosis, especially in astrocytes, a type of neuroglial cells [7]. Although the discussion on whether gliotransmission, a process depending on the rapid detection of synaptic activity by astrocytes, is present *in vivo*, ample evidences indicate that regulated exocytosis is present in astrocytes (Verkhratsky et al. EMBO J. 2016; 35: 239), however, it is much slower than in neurons (Kreft et al. Glia. 2004; 46:437). The paradigm of gliotransmission being present *in vivo*, critically depends on experiments in mice expressing the dnSNARE peptide. However, the mechanism of action of this peptide was only recently described [8]. In contrast to the previously considered mechanism, where the dnSNARE peptide interferes exclusively at the stage of the formation of the SNARE complex between the vesicles and the plasmalemma, the results revealed that the dnSNARE peptides strongly affect the properties of the fusion pore after the vesicle membrane merges with the plasmalemma. In the cells with overexpressed neurotoxins, which cleave the SNARE proteins, the frequency of unitary exocytotic events was reduced, as is expected, if the formation of ternary SNARE complex formation is required for the vesicle membrane merger with the plasmalemma. In contrast, however, in the presence of the dnSNARE peptide, the frequency of exocytotic events was unaffected. Moreover, in the dnSNARE peptide-treated cells transient fusion pores exhibited narrow diameters that could not widen or even transit to full-fusion upon stimulation [8]. The dnSNARE-mediated fusion pore stabilization [8], which is considered release unproductive, was similar to the stabilization observed in lactotrophs transfected with Munc 18-1 mutants [6]. Together these studies revealed that the ternary SNARE complex assembly/disassembly may affect the exocytotic fusion pore at multiple intermediates between the narrow fusion pore formation and the transit of this stage into the full-fusion stage.
